# Self-protected nitrate reducing culture for intrinsic repair of concrete cracks

**DOI:** 10.3389/fmicb.2015.01228

**Published:** 2015-11-04

**Authors:** Yusuf Ç. Erşan, Elke Gruyaert, Ghislain Louis, Christine Lors, Nele De Belie, Nico Boon

**Affiliations:** ^1^Laboratory of Microbial Ecology and Technology, Department of Biochemical and Microbial Technology, Ghent UniversityGhent, Belgium; ^2^Magnel Laboratory for Concrete Research, Department of Structural Engineering, Ghent UniversityGhent, Belgium; ^3^Département Génie Civil et Environnemental, Ecole Nationale Supérieure des Mines de DouaiDouai, France

**Keywords:** self-encapsulated, self-healing, microbial concrete, indentation, mechanical properties, denitrifying culture, admixtures

## Abstract

Attentive monitoring and regular repair of concrete cracks are necessary to avoid further durability problems. As an alternative to current maintenance methods, intrinsic repair systems which enable self-healing of cracks have been investigated. Exploiting microbial induced CaCO_3_ precipitation (MICP) using (protected) axenic cultures is one of the proposed methods. Yet, only a few of the suggested healing agents were economically feasible for *in situ* application. This study presents a ${\text{NO}}_{3}^{-}$ reducing self-protected enrichment culture as a self-healing additive for concrete. Concrete admixtures Ca(NO_3_)_2_ and Ca(HCOO)_2_ were used as nutrients. The enrichment culture, grown as granules (0.5–2 mm) consisting of 70% biomass and 30% inorganic salts were added into mortar without any additional protection. Upon 28 days curing, mortar specimens were subjected to direct tensile load and multiple cracks (0.1–0.6 mm) were achieved. Cracked specimens were immersed in water for 28 days and effective crack closure up to 0.5 mm crack width was achieved through calcite precipitation. Microbial activity during crack healing was monitored through weekly NOx analysis which revealed that 92 ± 2% of the available ${\text{NO}}_{3}^{-}$ was consumed. Another set of specimens were cracked after 6 months curing, thus the effect of curing time on healing efficiency was investigated, and mineral formation at the inner crack surfaces was observed, resulting in 70% less capillary water absorption compared to healed control specimens. In conclusion, enriched mixed denitrifying cultures structured in self-protecting granules are very promising strategies to enhance microbial self-healing.

## Introduction

Cracking of concrete is inevitable due to its heterogeneous matrix and brittle nature. Early age cracks in concrete mostly occur a few days after casting and facilitate the migration of aggressive substances toward the steel reinforcement. Microbial induced CaCO_3_ precipitation (MICP) became a popular research topic and an effective strategy for autonomous healing of concrete cracks. As described so far, typical components of a healing agent in microbial self-healing concrete are the bacterial agent, a protective carrier and the necessary nutrients for stimulation of bacterial activity (Wiktor and Jonkers, 2011; Achal et al., 2013; Wang et al., 2014a,b). Recent advances in bacteria-based self-healing concrete brought the technology closer to application. For instance, in 2014, a large scale application of self-healing concrete took place in Ecuador (Sierra-Beltran et al., 2015), yet the performance is unknown. Axenic cultures having specific traits are the main interest as bacterial agents by now (Wiktor and Jonkers, 2011; Achal et al., 2013; Wang et al., 2014a,b). On the one hand, using an axenic culture is important to evidence the potential of proposed microbial pathways for their use in microbial self-healing concrete. Moreover, the use of axenic cultures eases the control of the process and minimizes unexpected results. On the other hand, using either of the currently proposed axenic strains as bacterial agents results in a considerable increase in the cost of the product (Silva et al., 2015a). Several options can be considered to decrease the cost of the healing agent (protected bacteria+nutrients) such as using commercially available protective carriers instead of microcapsules, or decreasing the amount of bacterial agent used depending on the expectations of the manufacturer. Differently, replacing axenic cultures by non-axenic cultures that are derived from side-streams can be a feasible alternative. In a recent study, Silva et al. (2015b) revealed that non-axenic cultures can also be produced to follow a single pathway by applying selective processes and thus the production cost can be decreased 40 times. Furthermore, in terms of the provided self-healing performance up to 400 μm crack width, the non-axenic ureolytic powder appeared to be as effective as *Bacillus sphaericus* which is one of the most popular axenic strains used in self-healing concrete studies (Wang et al., 2014a; Silva et al., 2015b). Moreover, the culture is reported to be a self-protected culture avoiding use of protective carriers for concrete application (Erşan et al., 2015c; Silva et al., 2015b). Since the concrete industry demands inexpensive solutions for durability issues, use of self-protected non-axenic cultures can pave the way for application of microbial self-healing concrete.

Apart from ureolysis, aerobic respiration and anoxic oxidation of organic carbon through ${\text{NO}}_{3}^{-}$ reduction are the two other pathways that are proven to be useful for development of microbial self-healing concrete (Wiktor and Jonkers, 2011; Erşan et al., 2015b). Wiktor and Jonkers (2011) reported crack closure up to 460 μm in 100 days for mortar specimens containing bacteria loaded light-weight aggregates. It is also known that the ${\text{NO}}_{3}^{-}$ reduction pathway leads to CaCO_3_ precipitation and in our previous work CaCO_3_ yields up to 18.9 g CaCO_3_/g NO_3_-N were achieved by only using the concrete admixtures Ca(HCOO)_2_ and Ca(NO_3_)_2_ as nutrients (Erşan et al., 2015a). Moreover, ${\text{NO}}_{3}^{-}$ reducing axenic strains could induce closure of concrete cracks up to 400 μm in 28 days (Erşan et al., 2015b). However, studies investigating these pathways are limited to use of axenic cultures. Therefore, it is necessary to produce and test self-protected non-axenic cultures that are able to follow the ${\text{NO}}_{3}^{-}$ reduction pathway for development of microbial self-healing concrete.

Granular bacterial cultures can be an option for use of non-axenic cultures in concrete. One of the main advantages of granulated cultures is the systematic placement of cultures in a compact form (Gao et al., 2011). For instance granules can consist of aerobic heterotrophs, denitrifiers, poly-phosphate accumulating bacteria, and nitrifiers at the same time (Gao et al., 2011). Depending on the cultivation process, selective enrichment of the certain type of species in a granulated culture is possible. Moreover, the compact form and the layered structure of granular biomass is advantageous for protection of the bacteria at the core (Erşan and Erguder, 2014). Studies revealed that granulated bacteria can be dried, stored, and re-activated in case of necessity (Lv et al., 2013). Therefore, it is possible to achieve a self-protected nitrate reducing community for concrete application by using the granulation phenomenon.

For such need, special granules called “activated compact denitrifying core” (ACDC) were cultivated in this study. Previous investigations revealed that ACDC can survive mortar incorporation, inhibits steel corrosion and is compatible with concrete (Erşan et al., 2015c, accepted). Yet, the self-healing performances of mortar specimens containing ACDC granules have not been tested. Therefore, it is necessary to investigate ACDC for development of microbial self-healing concrete. Furthermore, to our knowledge, microbial self-healing concrete studies are mostly limited to curing periods up to 56 days (Wiktor and Jonkers, 2011; Wang et al., 2014a,c) which is far from the concrete ages *in situ*. Thus, the self-healing performance of bacteria-based concrete at the long term is still a question. Accordingly the study was conducted in two consecutive steps (1) assessing the self-healing performance of mortar specimens containing ACDC, (2) assessing the self-healing performance of concrete cracks occurring after 6 months curing.

## Materials and methods

### Cultivation of self-protected granular culture

ACDC granules were cultivated in a cylindrical sequencing batch reactor (SBR) (effective *h* = 30 cm, Ø = 12.5 cm and 50% volume exchange ratio) by following a previously described procedure (Erşan and Erguder, 2013). The SBR was operated with anoxic/aerobic period sequence (180 min anoxic and 155–168 min aerobic period). Since the aim was to use the granules for concrete application, the granules should be composed of bacteria that are able to reduce ${\text{NO}}_{3}^{-}$ in the absence of micronutrients and vitamins. Therefore, different from the described procedure, minimal nutrient solution (COD:N—5:1) was used as feed (4 times/day) and the solution composition is given in Table 1. Moreover, the initial pH of the feed solution was set between 9 and 9.5 by using concentrated NaOH solution (10 M). Granulation [94% of the volatile suspended solid (VSS) content] was achieved in 4 weeks and the reactor was operated for 7 months in total. ACDC granules were harvested from the reactor at the end of 2nd, 4th, and 7th month. The harvesting times were arbitrarily chosen after achieving the stable granular biomass (~95%) with an active denitrifying core. The harvested ACDC granules were dried for 48 h in a drying tunnel at 60°C with ventilation and stored at room temperature until the tests.

**Table 1:** **The feed composition for cultivation of ACDC**.

**Compounds**	**Concentrations (g/L)**
NaNO_3_	1.7
NaHCOO	5.36
Ca(HCOO)_2_	0.65
Na_2_HPO_4_.2H_2_O	0.06
MgSO_4_.7H_2_O	0.18

### Quality assessment of the culture

During the steady operation of the SBR, initial and effluent NOx-N (NO_3_-N and NO_2_-N) concentrations were monitored by sampling the arbitrary cycles. The rough composition of the ACDC granules was also monitored through volatile (VSS) and total suspended solid (TSS) analysis after full granulation (>90% granulation). Based on the VSS:TSS ratio, ACDC granules consisted of bacteria (0.7 w/w) and inorganic matter (0.3 w/w). Throughout the manuscript, ACDC amounts are given as cell dry weight (CDW) which represents 70% of the total ACDC amount used. The objective was to achieve granular culture containing an active denitrifying core. One of the indications of a denitrifying core is denitrification activity in the aerobic period (Erşan and Erguder, 2014) when the dissolved oxygen concentration is around 6 mg/L. Therefore, the activity of the core community was monitored through kinetic NOx-N measurements with 15 min intervals during the aerobic period of a cycle.

### Preparation of the mortar specimens and formation of the cracks

Series of mortar specimens (30 × 30 × 360 mm) with an embedded steel reinforcement bar (Ø = 6 mm) were prepared by using CEM I 52.5 N, tap water and standard sand according to the norm EN 196-1 and further cured at 20°C and RH > 90% for 28 days and 6 months prior to cracking. The sand:cement:water ratio was 3:1:0.5. Self-healing additives consisted of nutrients and self-protected bacterial agent. Commercial concrete admixtures calcium formate [Ca(HCOO)_2_ – 2% w/w cement] and calcium nitrate [Ca(NO_3_)_2_ – 3% w/w cement] were used as nutrients. Dry ACDC granules (0.5–2 mm in size) were used as bacterial agent and added into the mix during the mortar preparation. Sieving technique was used to achieve the portion with the desired size range. The influence of the initial amount of the bacterial agent was tested by using two different doses (either 0.5 or 1% w/w cement).

Cured specimens (at 20°C and RH > 90%) were subjected to a tensile stress by applying uniaxial tensile load at a speed of 0.01 mm/s on the embedded steel reinforcement bar under stroke control. Multiple cracks were achieved and the load was increased until a desired average crack width was achieved. The average crack width was calculated as previously described (Wang et al., 2014c).

The mortar specimens cured for 28 days were tested for 100–600 μm crack width range. The mortar specimens cured for 6 months were tested for 100–500 μm crack width. Average crack widths of each series are given in Table 2. Following the cracking, specimens were immersed in water for 28 days at ambient temperature of 20°C.

**Table 2:** **Detailed information about the specimen series tested throughout the study**.

**Series**	**Bacteria dose (% w/w cement)**	**Crack width range (μm)**	**Average crack width (μm ± SEM)**	**Age (curing time)**
Reference	N/A	65–490	250±9	28 days
	N/A	100–500	280±5	6 months
Abiotic Control (R+N)	N/A	65–530	270±10	28 days
	N/A	120–500	260±5	6 months
Microbial Specimen (R+N+ACDC)^a^	0.5	100–640	400±10	28 days
	1	90–640	350±10	28 days
	0.5	130–550	320±7	6 months
	1	120–500	310±5	6 months

a *R, Reference mortar [sand:cement:water (g)- 1350:450:225]; N, Nutrients; ACDC, Activated Compact Denitrifying Core; N/A, No addition*.

### Quantification of self-healing properties

#### Crack closure

Crack closure was observed biweekly through stereomicroscope with Leica S8 Apo apochromatic optics (Diegem, Belgium). Obtained images were further analyzed for the decrease in crack width by using image analysis software (The Leica Application Suite, LAS 3.7). During microscopic analysis, specimens exposed to ambient air conditions (~20°C). Crack closure efficiency was calculated by using Equation (1).

(1) $$\begin{array}{r} \text{Crackclosure \%}=\left[1-\left({\text{w}}_{\text{t}}∕{\text{w}}_{\text{initial}}\right)\right]\times 100 \end{array}$$

where, w_t_, crack width measured at a certain time t(d); w_initial_, initial crack width.

#### Water tightness

In order to quantify the water tightness of the healed specimens capillary sorption tests were conducted as described previously (Wang, 2013). Prior to testing, the specimens were dried in an oven at 40°C until the mass changes in 24 h were less than 0.1%. Similar crack widths were chosen for each specimen. Apart from the chosen crack the rest of the specimen was completely covered with aluminum tape to prevent water ingress and evaporation. Therefore, only the area of 3 cm^2^ (30 × 10 mm) surrounding the chosen crack contacted with water. The mass increase of specimens due to the absorbed water was monitored in regular time intervals. A wet towel was used to remove the remaining surface water droplets prior to weighing. Water tightness regain was calculated by considering the water tightness of the uncracked specimen as a goal of 100% regain and the water tightness of the autogenously healed specimen as a reference. The calculations were done by following Equation (2).

(2) $$\begin{array}{l} \text{Water tightness regain }\left(\%\right)=\\ \;\left(1-\frac{{\text{m}}_{\text{healed}}-{\text{m}}_{\text{uncracked}}}{{\text{m}}_{\text{autogenously healed}}-{\text{m}}_{\text{uncracked}}}\right)\;\times \;100 \end{array}$$

where, m_autogenouslyhealed_, water absorbed after autogenous healing; m_healed_, water absorbed after any type of healing; m_uncracked_, water absorbed by uncracked specimen.

### Mechanical and chemical characteristics of the healing material

A representative mortar slice was carefully sawn from each mortar bar by leaving ~5 mm distance from the crack borders and prepared for further testing. The samples (~10 × 25 × 25 mm) were embedded in Wood's alloy at their side surfaces and in epoxy resin at their top surface. The epoxy could not penetrate into the pores of the mortar specimen, only into the crack, and thus did not interfere with the mechanical properties of the calcite and C-S-H whilst indentation measurements. Surface quality is of importance for accurate determination of elastic moduli and hardness by indentation. Therefore, the specimens were gently ground using diamond grinding discs (grade 80, 220, 600, and 1200) and polished using diamond suspensions (6, 3, and 1 μm) in order to obtain a smooth surface. Finally, a carbon coating was applied and the specimens were vacuum-dried before investigation.

A Hitachi S-4300SE/N SEM (Berkshire, United Kingdom), equipped with a special stage to combine the indentation technique with SEM/EDX, was used during the investigation of mechanical properties. Such a combined system was beneficial to have certainty about the test location and the mineral phase for which the mechanical properties were measured and calculated. Moreover, the chemical composition of the products formed in the crack could be verified by energy dispersive X-ray analyses (EDX). During the indentation tests, the samples were visualized in the SEM with an accelerating voltage of 10 kV and a working distance of 35 mm. Furthermore, images with higher resolution were obtained at an acceleration voltage of 15 kV and a working distance of 8–10 mm. The micrographs shown for the 28 days old specimens were obtained with the latter settings.

The indentation tests were performed using the micro-hardness tester of the company Kammrath and Weiss (Dortmund, Germany). The load and displacement of the diamond Berkovich indenter were monitored with an accuracy of 0.5 mN and 1 nm, respectively. The displacement of the indenter was determined by means of a laser, which reflects on the Wood's alloy and allows to measure very accurately the penetration of the indenter in the matrix. Moreover, the displacement values were corrected to take into account the compliance of the test frame. Tests on cement-based materials showed that the value for the compliance C_*f*_ of the used test frame is 0.000561 μm/mN. The load cycle used for the indentation tests is given in Supplementary Figure 1. The load was increased to 25 mN at a speed of 0.8 mN/s and held constant for 15 s to avoid plastic effects. Thereafter, the load was decreased at the same speed (See Supplementary Figure 1). Based on the curve presenting the load in function of the penetration depth of the indenter (See Supplementary Figure 2), the hardness could be determined without visualization of the indentation.

As the maximum penetration was always more than 400 nm (Chicot, 2009) the measurements were situated at the micro-hardness level and the Martens hardness H_M_ [GPa] had to be calculated as the ratio of the maximum load P_max_ [mN] to the contact surface area A_c_ [μm^2^] Equation (3). For a Berkovich indenter, A_c_ can be calculated in function of h_c_ as given by Equation (4). Equation (4) is based on the fact that the behavior of the Berkovich indenter can be modeled by a conical indenter with a half-included angle of 70.3°, giving the same depth-to-area relationship (Oliver and Pharr, 2004). h_c_ [μm] is the contact depth as used in the calculation of the hardness by Oliver and Pharr (Oliver and Pharr, 1992) and is calculated based on the maximum penetration depth h_max_ [μm] (See Supplementary Figure 2).

(3) $$\begin{array}{rcl} {\text{H}}_{\text{M}} & = & \frac{{\text{P}}_{\text{max}}}{{\text{A}}_{\text{C}}} \end{array}$$

(4) $$\begin{array}{rcl} {\text{A}}_{\text{C}} & = & 26.43\cdot {\text{h}}_{\text{c}}^{2} \end{array}$$

(5) $$\begin{array}{rcl} {\text{h}}_{\text{c}} & = & {\text{h}}_{\text{max}}-\varepsilon \cdot \frac{{\text{P}}_{\text{max}}}{{\text{S}}_{\text{u}}}\text{ with }{\text{S}}_{\text{u}}={\left(\frac{\text{dP}}{\text{dh}}\right)}_{{\text{h}=\text{h}}_{\text{max}}} \end{array}$$

where, P_max_, the maximum load (mN); S_u_, the slope at the deloading curve in h = h_max_ (mN/μm); ε, the geometrical constant equals to 0.75 according to Oliver and Pharr (2004).

An approximate estimation of the E-modulus could be made based on the results obtained in this study. Therefore, the reduced modulus of elasticity (E_R_) [GPa] was first calculated with Equation (6), taking into account Equation (7).

(6) $$\begin{array}{rcl} \frac{1}{{\text{S}}_{\text{u}}} & = & {\left(\frac{\text{dh}}{\text{dP}}\right)}_{\text{h}=\text{hmax}}={\text{C}}_{\text{f}}+\frac{\sqrt{\pi }}{2}\cdot \frac{1}{\beta \cdot \lambda \cdot {\text{E}}_{\text{R}}}\cdot \frac{1}{\sqrt{{\text{A}}_{\text{CP}}}} \end{array}$$

(7) $$\begin{array}{rcl} {\text{A}}_{\text{CP}} & = & 24.5\cdot {\text{h}}_{\text{c}}^{2} \end{array}$$

where, C_f_, compliance value for the used test frame which equals to 0.000561 μm/mN; E_R_, modulus of elasticity; β and λ, correction factors which equal to 1.05 (Oliver and Pharr, 2004) and 1.1 (Hay et al., 2008), respectively; A_cP_, the projected contact area (μm^2^) as previously reported (Oliver and Pharr, 1992); h_c_, the depth (μm) as previously reported (Oliver and Pharr, 1992).

The E-modulus of the tested material (E_*m*_) is then calculated with Equation (8).

(8) $$\begin{array}{r} \frac{1}{{\text{E}}_{\text{r}}}=\frac{1-\nu_{\text{m}}^{2}}{{\text{E}}_{\text{m}}}+\frac{1-\nu_{\text{i}}^{2}}{{\text{E}}_{\text{i}}} \end{array}$$

where, ν_m_, estimated value for the Poisson coefficient of the tested cement based material which equals to 0.3; ν_i_, the Poisson coefficient of the diamond indenter which equals to 0.07; E_*i*_, the E-modulus of the diamond indenter which equals to 1140 GPa.

The mechanical characteristics of the original cement paste and the CaCO_3_ precipitated in the crack were determined by indentation tests. Ten to fifteen indentations were performed per test sample and per phase.

The inner crack surface of the mature specimens (6 months cured) was also analyzed, yet indentation tests were not conducted for these samples. During the visual inspection and EDX analysis of mature specimens, FEI Quanta 200F SEM/EDX (Oregon, USA) was used. In order to analyze and identify the healing materials inside the crack, a sawn sample with the crack (10 × 30 × 30 mm) was further split through the crack into two pieces by using manual force. One of the surfaces was coated with carbon (~15–35 nm thickness) and analyzed under SEM/EDX. The micrographs showing the inner crack surface of the mature specimens were taken at accelerating voltage of 15 kV and a working distance of 8–9 mm. The second piece was used for Fourier transform infrared spectroscopy (FTIR) analysis. The composition at a certain depth was the main interest, thus on the crack surface, an area of 1 cm^2^ (5 × 20 mm) was defined at a distance of 5 mm from the crack mouth and 2 mm from the reinforcement bar. The defined area was scraped by using a stainless steel spatula (5 mm width) and the pieces were collected (< 15 mg). Collected pieces were further ground into powder by using a mortar and pestle. A portion of the ground powder (< 5 mg) was chemically characterized by using Spectrum 100 FTIR (Perkim Elmer Inc, USA). Presented spectra were the result of 32 scans with a resolution of 4 cm^−1^ in the range of 4000–600 cm^−1^.

### Analytical methods

The VSS and TSS analysis were done according to the standard methods (APHA et al., 2012). Nitrate (${\text{NO}}_{3}^{-}$) and nitrite (${\text{NO}}_{2}^{-}$) concentrations were measured via compact Metrohm 761 ion chromatography (IC) (Herisau, Switzerland). Statistical analyses were conducted using SigmaPlot 12.0 (Systat Software Inc., USA) to compare significant differences by means of One way ANOVA test (*p* = 0.05).

## Results

### Quality assessment of the culture

**Figure 1:**
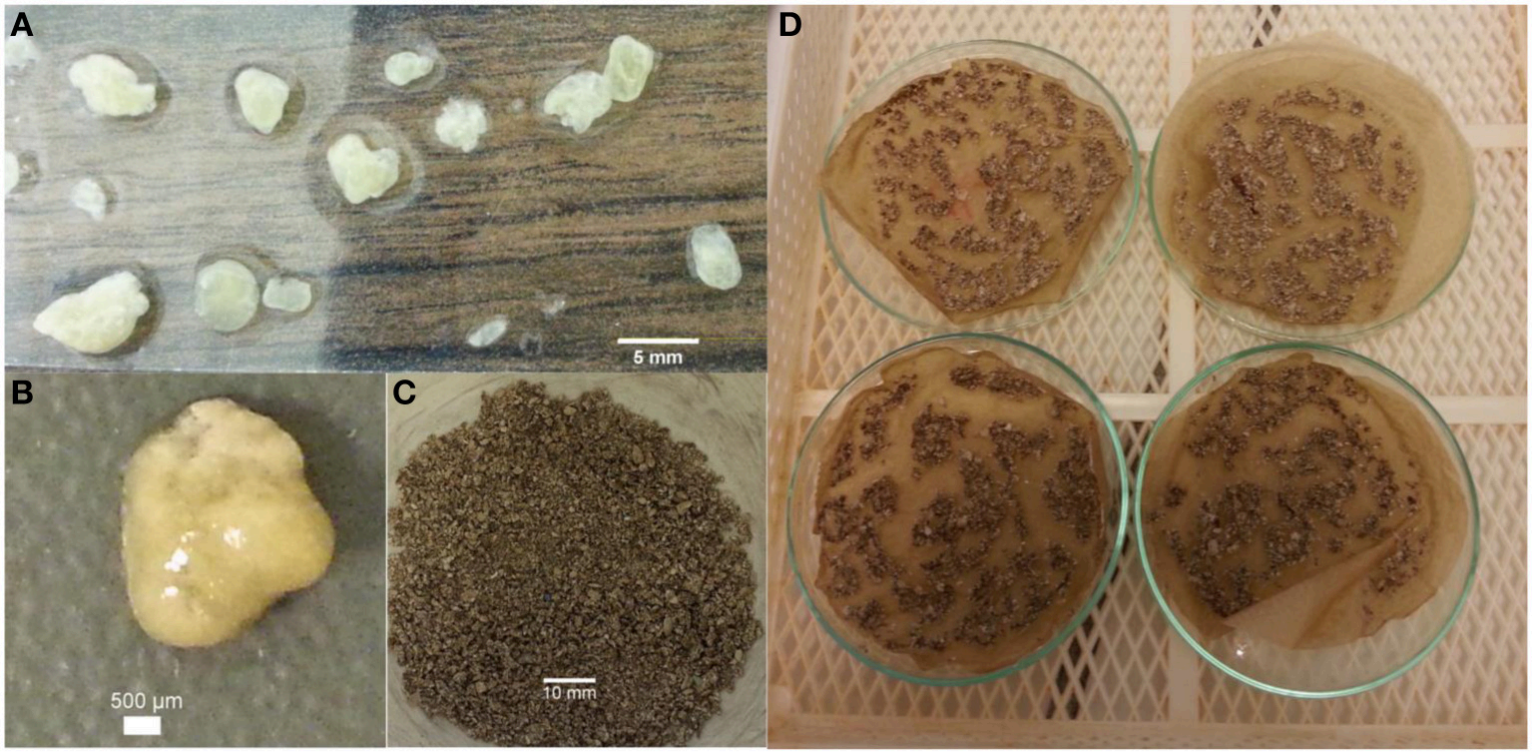
**Appearance of the harvested ACDC granules (A,B) wet; (C,D) after drying**.

The appearance of the harvested ACDC granules are given in Figures 1A,B. After the drying process, the ACDC granules shrink and their color became darker (Figures 1C,D). Based on the conducted solid analyses, the VSS concentration at steady-state operation was 5.5 ± 0.2 g/L and the TSS concentration was 7.8 ± 0.4 g/L.

**Figure 2:**
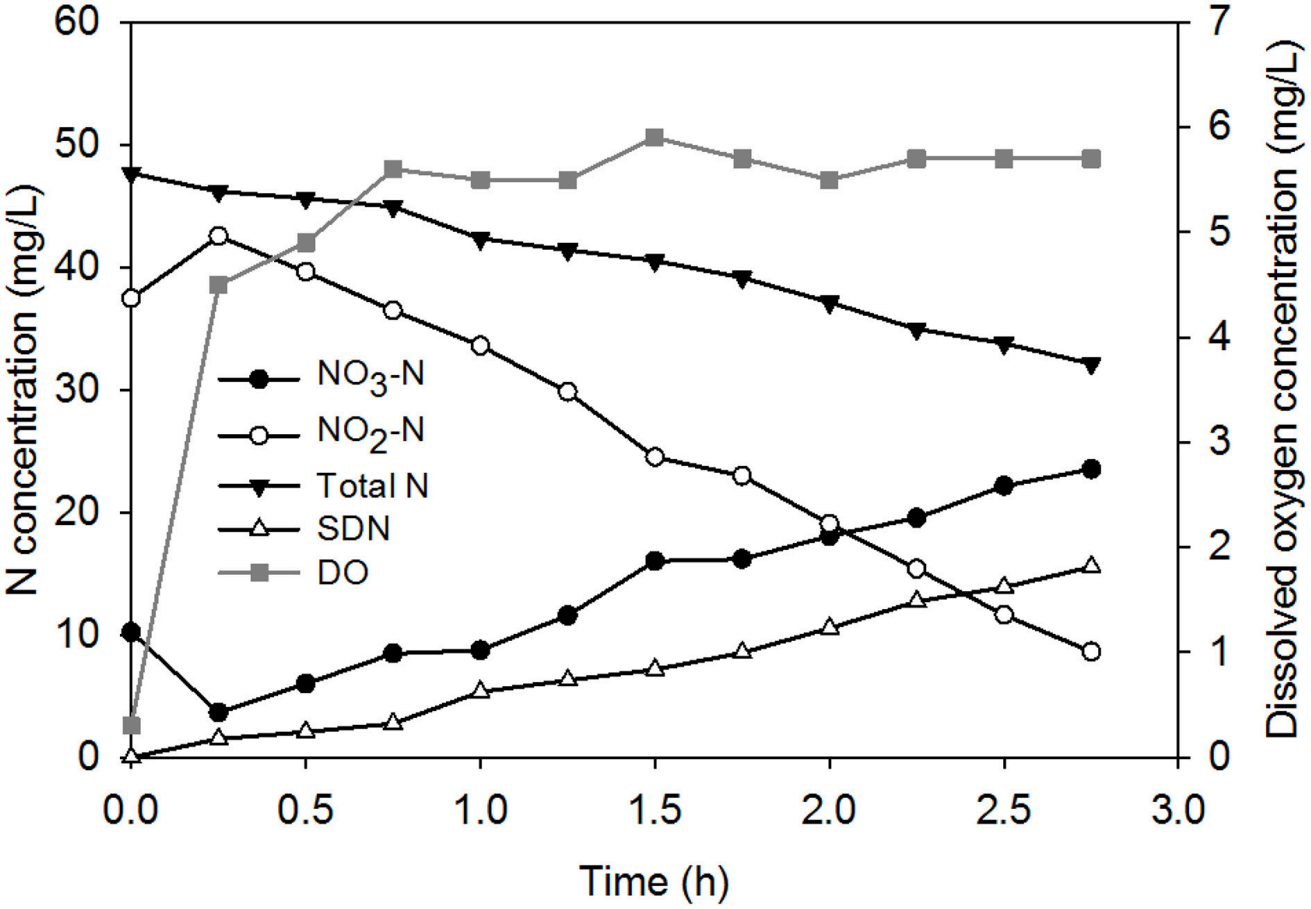
**Kinetic NOx-N measurements during the aerobic period of an arbitrarily chosen SBR cycle (SDN, simultaneous denitrification; DO, dissolved oxygen concentration)**.

At steady operation, ACDC:VSS ratio (the amount of granulated culture) in the system was more than 90%. The only nitrogen (N) source fed to the system was NO_3_-N. When the influent NO_3_-N concentration (280 mg/L, Table 1), the volume exchange ratio (50%) and the residual NOx-N (~32 mg/L, Figure 2) concentration at the end of a cycle during steady operation were taken into consideration, one can calculate the denitrification performance of ACDC for both anoxic and aerobic periods. Regular NOx-N measurements revealed that at the end of anoxic period 69% of the total nitrogen (TN) was consumed. Following kinetic NOx-N measurements in aerobic period (dissolved oxygen ~6mg/L), it appeared that there was simultaneous nitrification and denitrification. Results indicated that 34% of the TN was consumed through simultaneous denitrification occurring in aerobic period (Figure 2).

### Self-healing performance of microbial mortars containing ACDC culture

**Figure 3:**
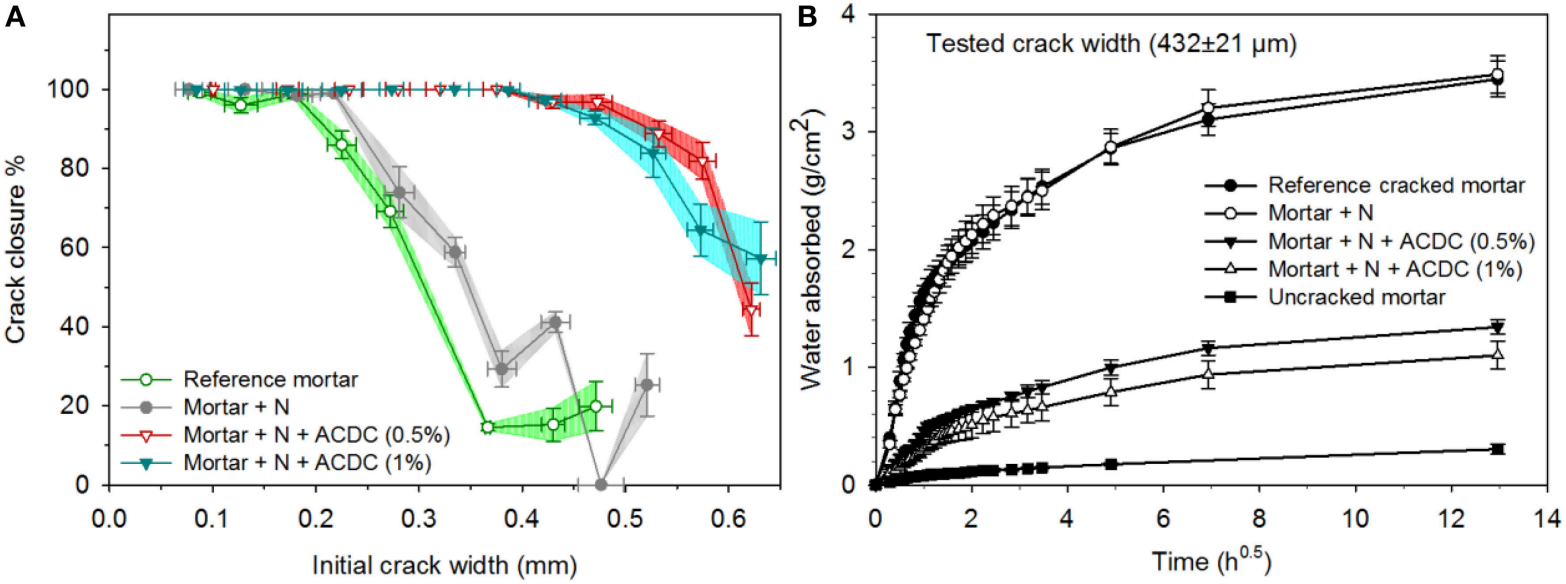
**The enhanced self-healing performance of the 28 days old microbial specimens over the control specimens (A) the crack closure performance (horizontal error bars represent the standard deviation, crack widths were grouped with 50 μm intervals, vertical error bars represent the standard error of the mean, *n* ≥ 5) (B) capillary sorption around the crack zone of the healed specimens [N: Nutrients – 2% Ca(HCOO)_2_ + 3% Ca(NO_3_)_2_; the error bars represent the standard deviation, *n* = 3]**.

**Figure 4:**
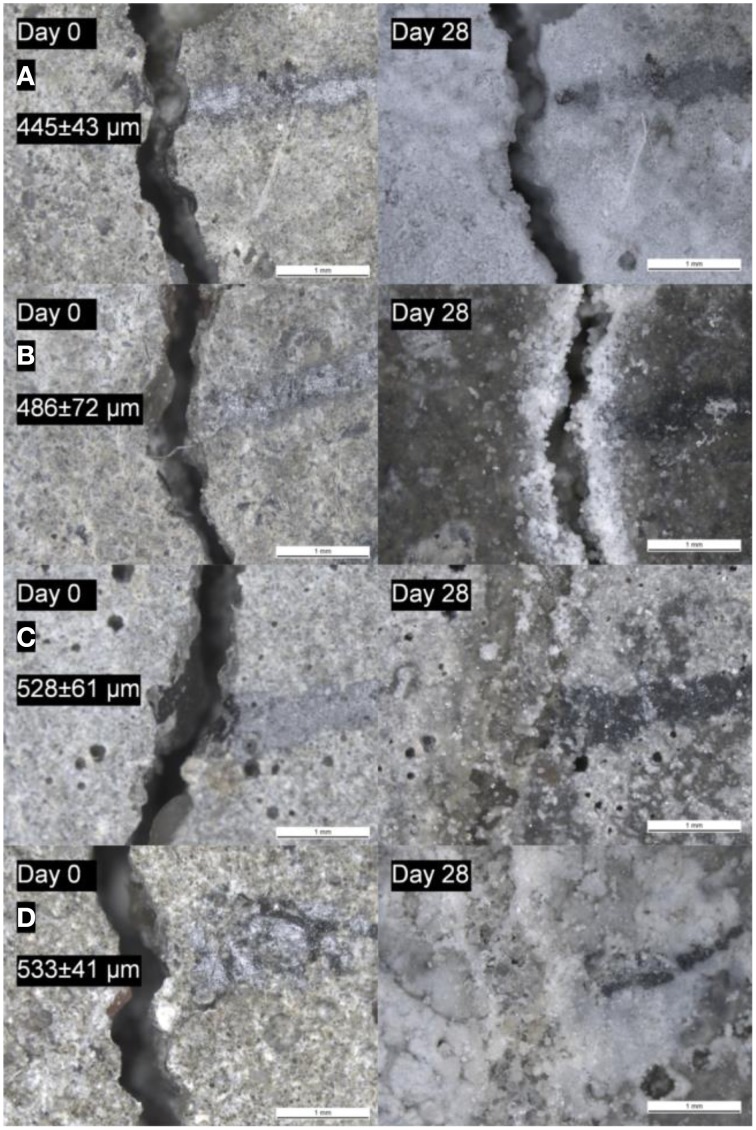
**The micrographs showing the initial (before incubation) and the final (after 28 days incubation) appearance of the cracks of the 28 days old specimens (A) reference specimen (B) abiotic control (reference + 5% nutrients) (C) specimen with 0.5% ACDC and 5% nutrients (D) specimen with 1% ACDC and 5% nutrients [N: Nutrients – 2% Ca(HCOO)_2_ + 3% Ca(NO_3_)_2_; weight percentages are in terms of cement weight]**.

The addition of ACDC and nutrients [concrete admixtures Ca(HCOO)_2_ and Ca(NO_3_)_2_] significantly improved the self-healing potential of mortar specimens (Figures 3A, 4). At the end of 28 days immersion in water, self-healing performances of the mortars containing 0.5% ACDC were similar to the ones containing 1% ACDC. Cracks up to 500 μm crack width were closed more than 90% (Figure 3A). The limit for the autogenous healing was recorded as 200 and 250 μm for reference specimen and abiotic control specimen, respectively (Figure 3A). Crack closure performances of the reference specimens sharply decreased when the initial crack widths were more than 200 μm. Among the control specimens, the ones containing nutrients (abiotic control) showed significantly better healing performance and could close cracks up to 250 μm (Figure 3A).

Capillary sorption tests revealed that microbial specimens could have a better water tightness than the control specimens (Figure 3B). The capillary sorption tests were conducted around the cracks with 432 ± 21 μm crack width for the specimens cracked at 28 days and healed for 28 days. Reference and abiotic control specimens were found to be similar in terms of water tightness. After the 28 days healing period, the microbial specimens containing 1 and 0.5% ACDC (w/w cement) absorbed 68 ± 5% and 61 ± 4% less water around the crack (~432 ± 21 μm initial crack width) than the reference specimens, respectively (Figure 3B). Different from the crack closure performances, in terms of water tightness, there was a slight but significant difference between the microbial specimens containing two different amounts of ACDC. Mortar specimens containing 1% ACDC absorbed 18 ± 8% less water than the mortar specimens containing 0.5% ACDC (Figure 3B).

### Nutrient availability and the microbial activity during incubation

**Figure 5:**
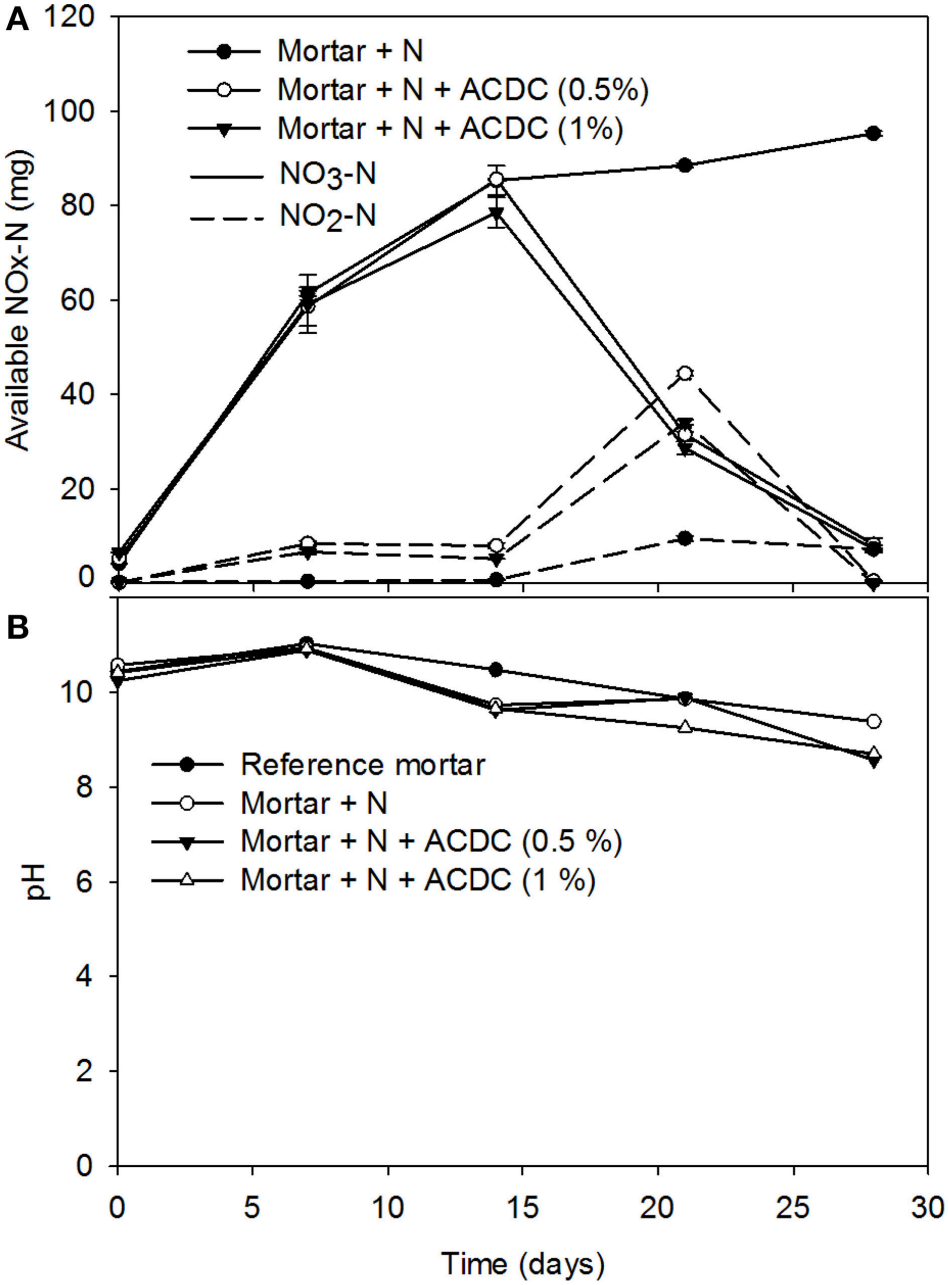
**Evolution of the available NOx-N in the bulk solutions during the 28 days incubation of the 28 days old specimens and the pH of the solution (A) mass of the available NOx-N (B) the pH change [N: Nutrients – 2% Ca(HCOO)_2_ + 3% Ca(NO_3_)_2_; *n* = 3, error bars represent the standard deviation]**.

One of the components of microbial self-healing concrete is the nutrients that initiate and maintain the bacterial activity. Therefore, availability of the nutrients for the bacteria is important. The results of the abiotic control are representative for the NOx-N passed from mortar to the solution, thus indicates the nutrient availability (Figure 5A). Based on the results, 14% of the NO_3_-N in a mortar specimen became available for microbial use (See Supplementary Material for calculations). Additionally, one can confirm the bacterial activity by the evolution of the abundance of nitrate or formate in solution. Weekly measurements of the NOx-N revealed that 92 ± 2% of the available NO_3_-N was consumed by the ACDC culture during the crack closure process (Figure 5A). Bacterial activity (${\text{NO}}_{3}^{-}$ reduction rate) significantly improved after pH decreased below 10 (Figures 5A,B).

### Mechanical properties of the healing material inside the crack

**Figure 6:**
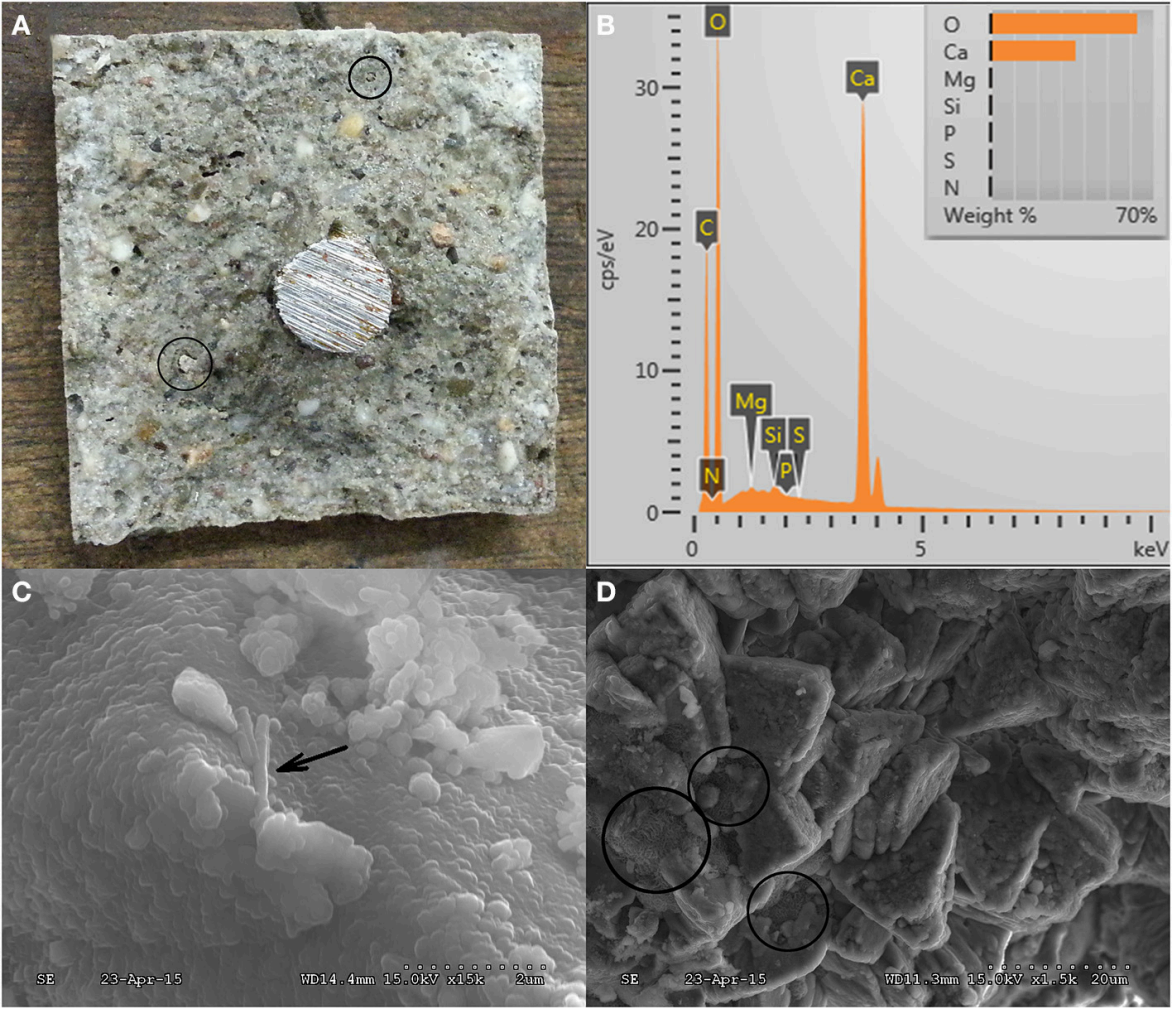
**Micrographs showing the inner crack surface of the 28 days old microbial specimen and the EDX results (A) inner crack surface appearance of microbial specimen after splitting (B) chemical composition of the minerals around the bacteria (C,D) SEM micrographs of the inner crack surface (“↗” indicates the individual bacterial remains, “°” indicates the calcified ACDC cluster)**.

The abundant formation on the inner crack surface of the microbial specimen was a white colored precipitate (Figure 6A). Visual inspection revealed that observed white precipitates were biochemically formed CaCO_3_ (in the form of calcite) (Figures 6C,D). Moreover, a portion of the ACDC granule (~40 μm wide) and the CaCO_3_ formation around the cluster could be visualized during the SEM analysis (Figure 6D). Additionally, individual bacterial remains were also found on precipitates (Figure 6C).

**Figure 7:**
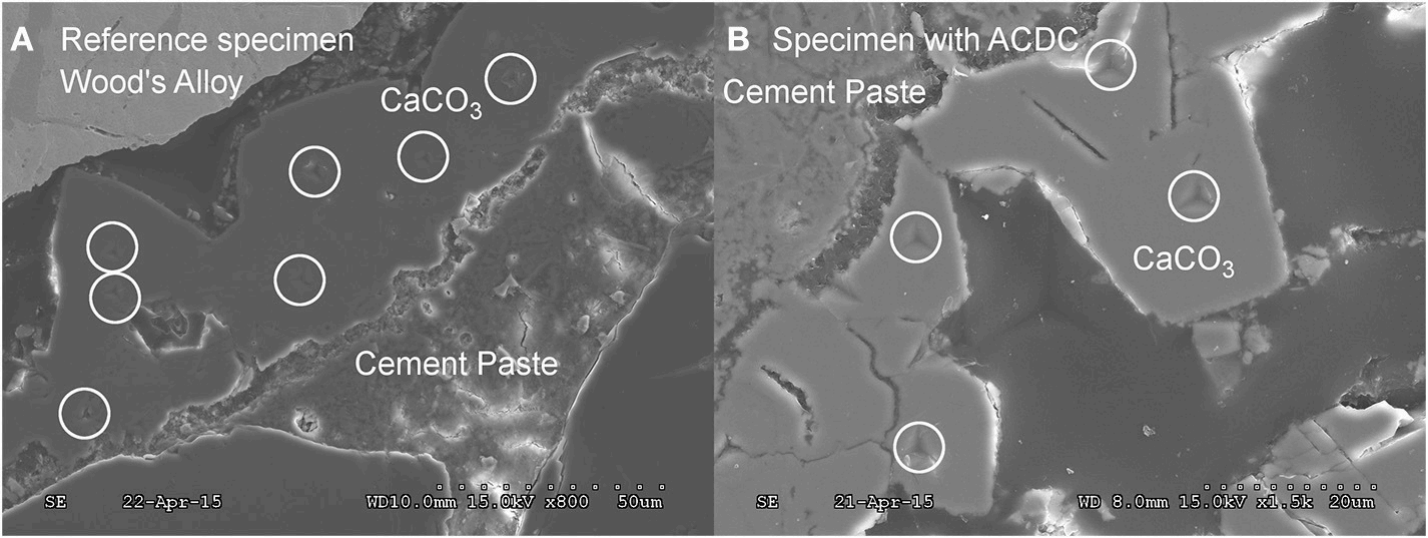
**SEM micrographs showing the indentation points whilst mechanical testing of the calcite (A) calcite formed in reference specimen (B) calcite formed in microbial specimen (0.5% ACDC)**. “°” indicates the points analyzed during indentation. Please refer to Supplementary Figure 3 for higher resolution on indentation.

**Figure 8:**
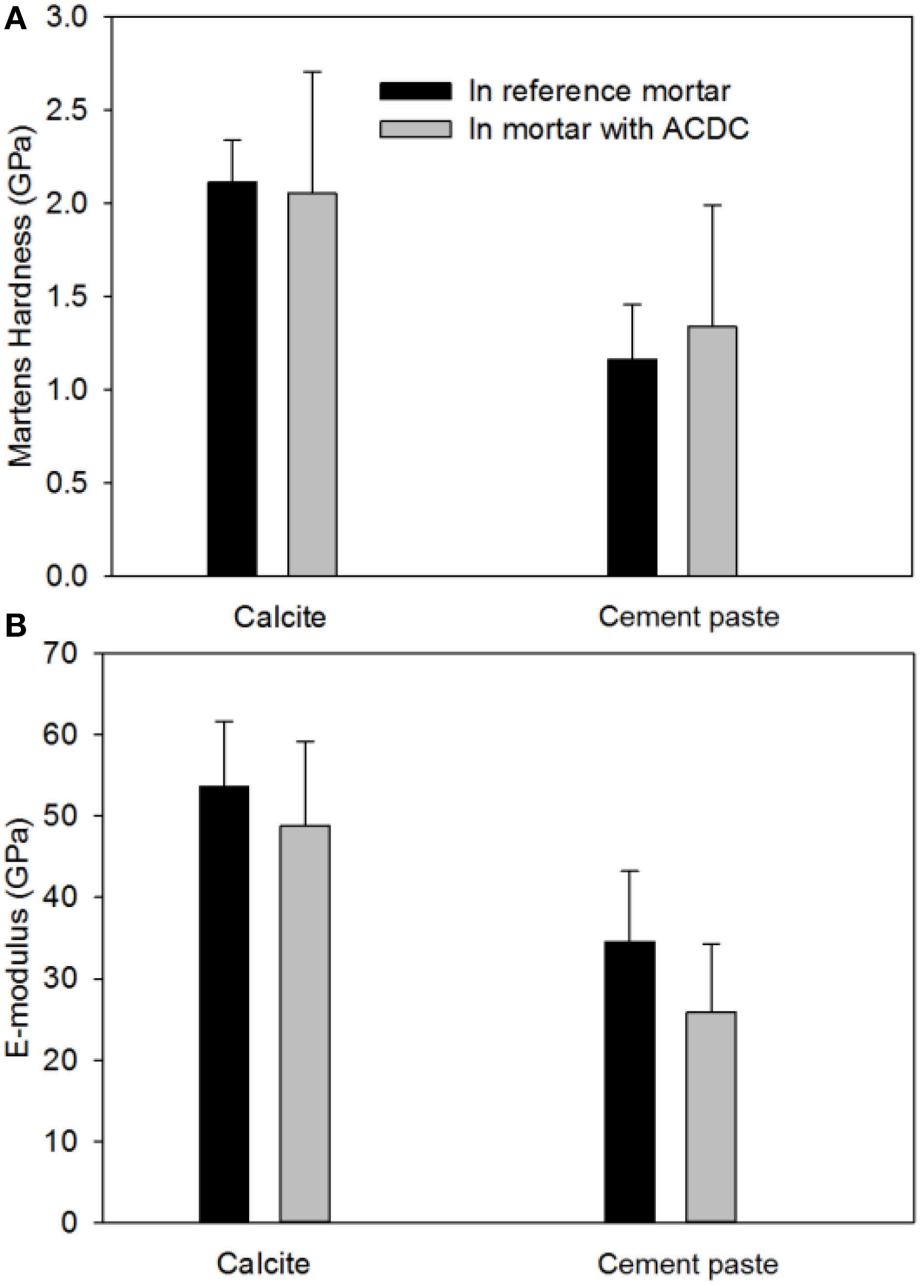
**Mechanical properties of the calcite and the cement paste found in autogenously and microbial healed specimens (A) Martens hardness values (B) calculated modulus of elasticity (*n* ≥ 10, error bars represent the standard deviation)**.

In addition to visual analysis, mechanical properties of the calcite crystals were quantified through indentation tests (Figure 7). Martens hardness was measured and based on the available data approximate E-modulus values were calculated. The Martens hardness values of 2.1 ± 0.2 GPa and 2.1 ± 0.7 GPa were achieved from the tested CaCO_3_ minerals in reference and microbial specimens, respectively (Figure 8A). The variation of hardness values for CaCO_3_ minerals were higher in microbial specimens than the reference specimens, yet they did not significantly differ among the specimens (Figure 8A). Further visual inspection of the indentation points revealed that on some of the minerals nano-cracks occurred under indentation load (See Supplementary Figure 3). These observed cracks were not particular for a certain type of specimen and observed in both cases. Following indentation tests, sampling points and the different layers were visualized. Indentation measurements were also conducted on hydrated cement paste. Martens hardness values of 1.2 ± 0.3 GPa and 1.3 ± 0.7 were obtained for the hydrated cement paste in reference and microbial samples, respectively (Figure 8A).

E-modulus values for the tested CaCO_3_ minerals were calculated as 54 ± 10 GPa and 49 ± 8 GPa from the data obtained whilst testing reference and microbial samples, respectively (Figure 8B). The E-modulus values for the hydrated cement paste were 35 ± 9 GPa and 26 ± 8 GPa for reference and microbial specimens (Figure 8B).

### Chemical characterization of the healing material

**Figure 9:**
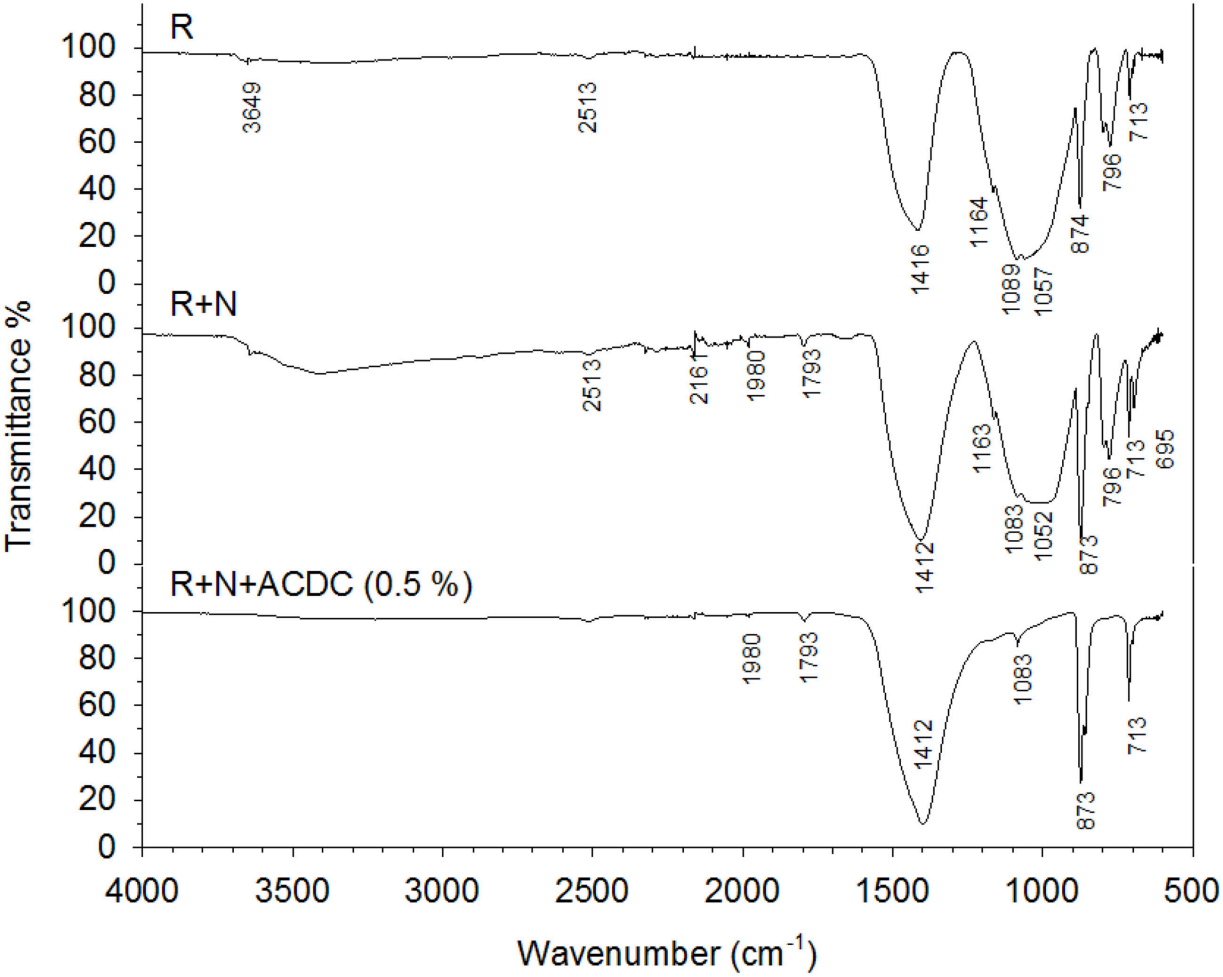
**FTIR spectra of the healing materials collected from the inner crack surfaces of 28 days old specimens [R, Reference; N, Nutrients – 2% Ca(HCOO)_2_ + 3% Ca(NO_3_)_2_; 5 mm < sampling depth < 13 mm]**.

In addition to the SEM imaging, the elemental composition of minerals was analyzed by using EDX spectroscopy. Visualized minerals were composed of Ca, C, and O elements which indicated that the minerals were most probably CaCO_3_ (Figure 6B). In order to confirm the EDX results, powders were collected from the counter face of the crack surface and chemically characterized via FTIR analysis. The information collected from both analyses revealed that the visualized and tested minerals inside the crack of microbial specimens were CaCO_3_ (Figures 6B, 9). In addition to calcite (2513, 1793, 1412, 873, and 713 cm^−1^), aragonite (696 cm^−1^), ettringite (1163 cm^−1^), bassanite (1083 cm^−1^), C_2_S (873 cm^−1^), C-S-H (1052, 1980, and 2163 cm^−1^), and portlandite (3649 cm^−1^) were found in the collected powder samples (Hughes et al., 1995; Mollah et al., 2000; Yu et al., 2004; Trezza et al., 2007) (Figure 9). In the powders collected from control specimens, cement and its hydration products were as abundant as the forms of CaCO_3_ (Figure 9) Contrarily, in the powder collected from the microbial specimens, different forms of CaCO_3_ were the dominant compounds (Figure 9).

### Effect of age on microbial crack healing

**Figure 10:**
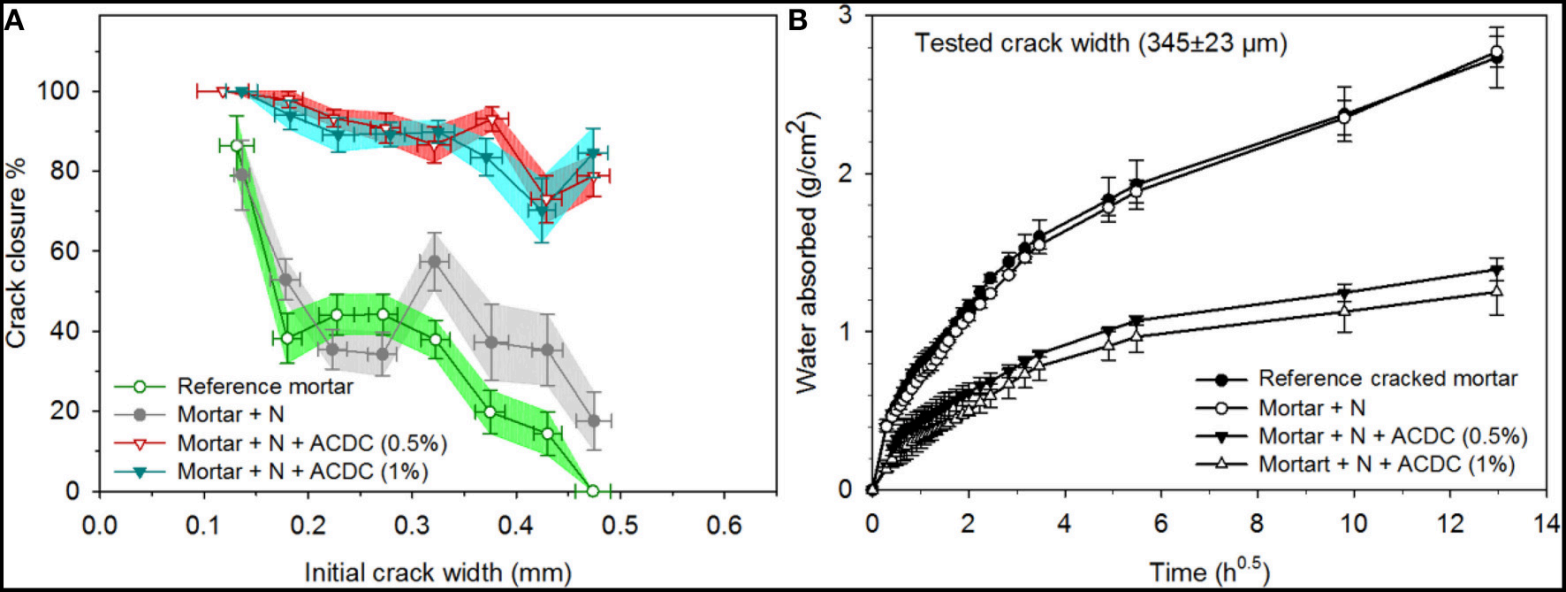
**The enhanced self-healing performance of the 6 months old microbial specimens over the control specimens (A) the crack closure performance (horizontal error bars represent the standard deviation, crack widths were grouped with 50 μm intervals, vertical error bars represent the standard error of the mean, *n* ≥ 5) (B) capillary sorption around the crack zone of the healed specimens [N: Nutrients – 2% Ca(HCOO)_2_ + 3% Ca(NO_3_)_2_; the error bars represent the standard deviation, *n* = 3]**.

**Figure 11:**
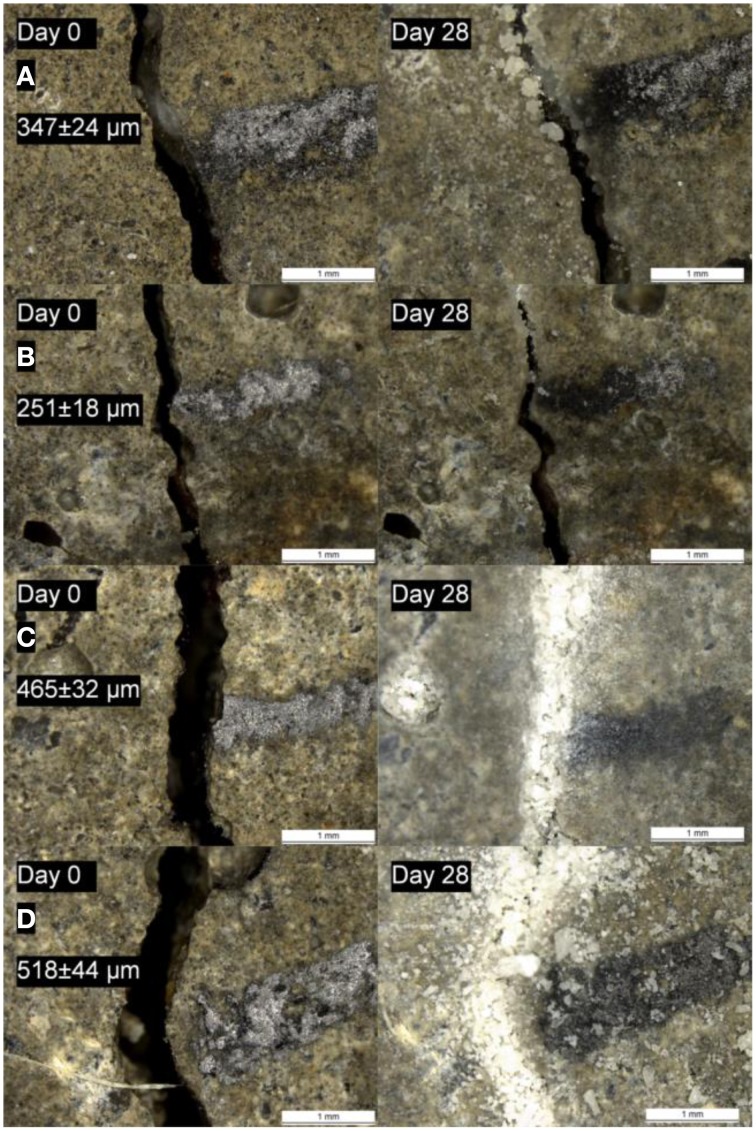
**The micrographs showing the initial (before incubation) and the final (after 28 days incubation) appearance of the cracks of the 6 months old specimens (A) reference specimen; (B) abiotic control (reference + 5% nutrients); (C) specimen with 0.5% ACDC and 5% nutrients; (D) specimen with 1% ACDC and 5% nutrients [Nutrients – 2% Ca(HCOO)_2_ + 3% Ca(NO_3_)_2_; weight percentages are in terms of cement weight]**.

Autogenous healing performance of the control specimens significantly decreased when the cracks formed after 6 months curing instead of 28 days curing (Figures 10A, 11). Based on the data obtained for the investigated crack width range (100–500 μm), autogenous healing performance after 6 months was recorded as 86 ± 8% for 135 μm crack width and gradually decreased with an increasing initial crack width (Figure 10A). Although, a noticeable decrease also appeared in microbial samples, ~90% crack closure was achieved for the cracks up to 400 μm crack width (Figure 10A). The closure performance of the cracks larger than 400 μm was still more than 70% (Figures 10A, 11).

Capillary sorption tests revealed that microbial specimens could have a better water tightness than the control specimens (Figure 10B). The capillary sorption tests were conducted around the cracks with 345 ± 23 μm crack width for 6 month old specimens. Reference and abiotic control specimens were found to be similar in terms of water tightness (Figure 10B). After the 28 days healing period, the microbial specimens containing 1 and 0.5% ACDC (w/w cement) absorbed 54 ± 8% and 49 ± 6 less water around the crack (~345 ± 23 μm initial crack width) than the reference specimens, respectively (Figure 10B). Among the microbial specimens, no significant differences were observed in terms of water tightness.

**Figure 12:**
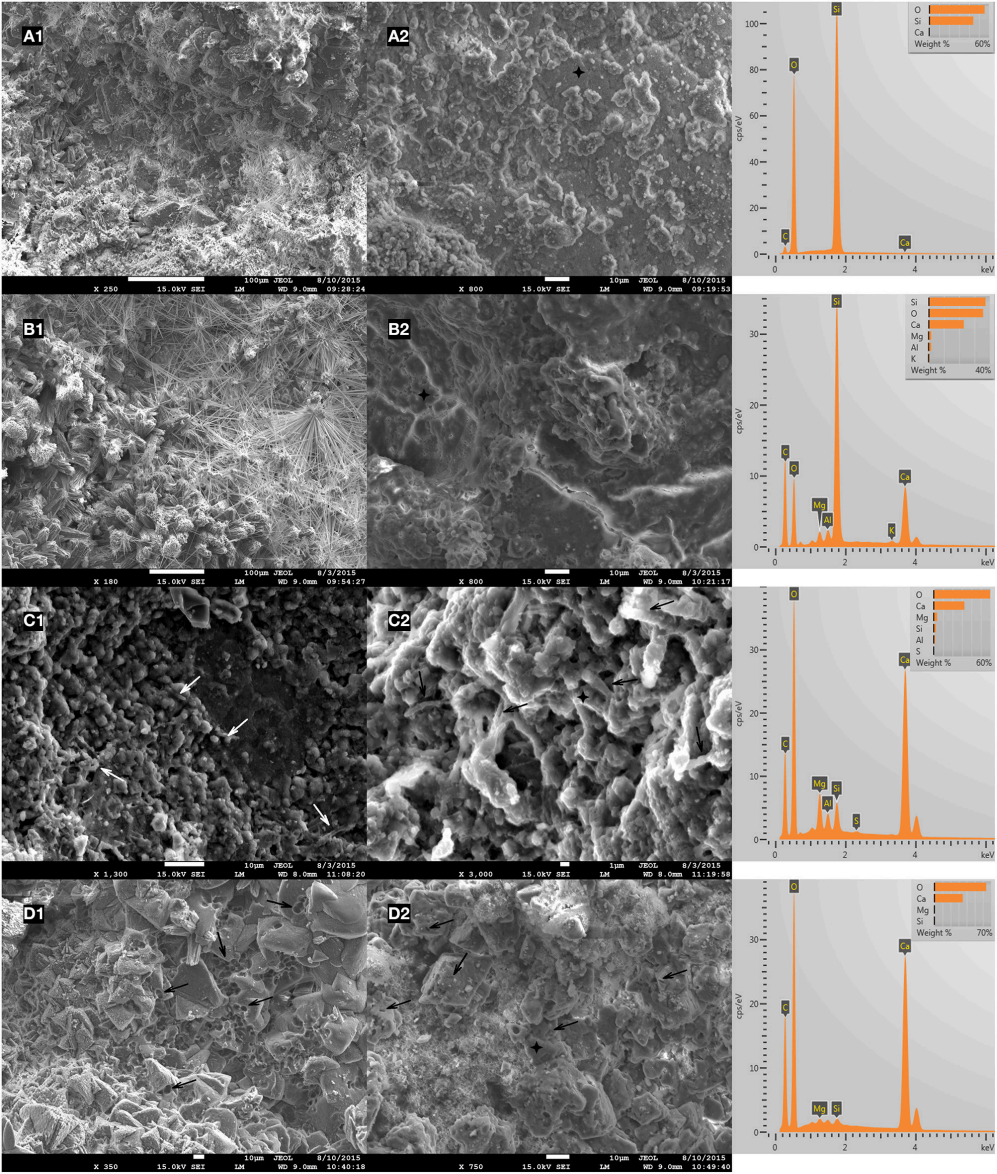
**Representative SEM micrographs and EDX analysis of the dominant healing material on the inner crack surface of 6 months old specimens after 28 days of healing (A1,A2) reference mortar; (B1,B2) abiotic control (reference + 5% nutrients); (C1,C2) mortar containing 0.5% ACDC and 5% nutrients; (D1,D2) mortar containing 1% ACDC and 5% nutrient [Nutrients – 2% Ca(HCOO)_2_ + 3% Ca(NO_3_)_2_; weight percentages are in terms of cement weight; “↗” indicates the calcified bacterial remains; “+” indicates the points analyzed via EDX]**.

The inner crack surface was visually explored in both control and microbial specimens. During the visual inspection of microbial specimens, a significant amount of biochemically formed CaCO_3_ minerals and calcified bacteria were found (Figures 12C,D). On the inner crack surface of the control specimens, CaCO_3_ and C-S-H were found (Figures 12A,B). Especially in the EDX spectrums obtained on the inner crack surface of control specimens, Si peaks were significant which was confirming the abundance of C-S-H (Figures 12A,B). Differently, the C and O peaks were remarkable in microbial specimens indicating the abundance of CaCO_3_ (Figures 12C,D). Moreover, some ettringite shaped formations were found in control specimens (Figures 12A,B).

## Discussion

### Quality of the ACDC

The denitrification performance achieved under aerobic conditions was an indication of a denitrifying core. The major reason of the denitrifying activity was the size of the granules that prevented oxygen diffusion creating an anoxic zone at the core (Erşan and Erguder, 2013). The anoxic/aerobic operation enhanced the enrichment of nitrate reducing bacteria at the core of the granules which was consistent with the previous studies (Wan et al., 2009; Erşan and Erguder, 2013). In addition to the denitrification, nitrification was also observed in the aerobic period which indicated the presence of nitrite oxidizing bacteria near the surface of the granules. This type of placement was an indication for the aimed layered structure. The inorganic content of the ACDC was 30% which was higher than the content of a typical granular biomass (Wan et al., 2009; Erşan and Erguder, 2013). This was due to the feed composition that was enriched in terms of Ca^2+^ (0.10 g/L Ca^2+^) compared to the synthetic feed composition used (contains 0.03 g/L Ca^2+^) in the reference study (Erşan and Erguder, 2013). The VSS/TSS ratio of 0.6 was reported for the anaerobic granules cultivated by using 0.15 g/L Ca^2+^ and increased to 0.7 when the influent Ca^2+^ concentration was 0.01 g/L (Yu et al., 2001). Previous studies also showed that the Ca^2+^ related precipitates mainly form the inorganic content of the granular biomass (Ren et al., 2008). Therefore, for ACDC, the major constituents of the inorganic content was expected to be CaCO_3_ and Ca(PO_4_)_3_ which also act as a protective layer for the nitrate reducers at the core.

### Crack closure and functionality regain

Autogenous healing limits of the 28 days old mortar specimens were recorded as 200 and 250 μm for reference and abiotic control, respectively. Previous studies reported comparable results for plain mortar specimens under similar incubation conditions (immersion in tap water) (Van Tittelboom and De Belie, 2013; Wang et al., 2014c; Silva et al., 2015b). The major reasons of autogenous healing were investigated in many studies and listed as (i) swelling of the crack walls, (ii) further hydration of unhydrated cement paste, (iii) blockage due to the sedimentation of fractured pieces, (iv) formation of CaCO_3_ at the crack mouth due to the carbonation of portlandite (Edvardsen, 1999; Van Tittelboom and De Belie, 2013).

Among the control specimens, abiotic control specimens showed slightly higher autogenous healing compared to the reference specimens. This enhanced performance could be due to the positive effect of admixtures on CaCO_3_ precipitation. CaCO_3_ precipitation is highly influenced by the pH, the Ca^2+^ concentration, the dissolved inorganic carbon concentration and the presence of nucleation sites. The abiotic control had higher Ca^2+^ concentration due to the addition of Ca(HCOO)_2_ and Ca(NO_3_)_2_ which might improve the CaCO_3_ precipitation and thus the crack closure performance of the abiotic control. Similar improvement was observed in previous studies when the Ca^2+^ concentration was increased (Wang et al., 2012a, 2014c).

The autogenous healing performance significantly decreased when the cracks formed in mature specimens (6 months old). It was not possible to effectively close the 150 μm crack width through autogenous healing. Yang et al. (2009), also suggested that the crack widths on mature specimens should be kept less than 150 μm which is consistent with our observation. These results indicated that the hydration of the unhydrated cement in pre-mature samples has significant influence on autogenous healing potential.

Unlike the abiotic control, microbial specimens considerably improved the self-healing potential for both early age and mature cracks. Although it is known that the spores can survive in extreme environments for a long time (Harwood and Cutting, 1990), the self-healing performance of a mature microbial concrete was always a concern. In this study, the crack closure performance of a mature microbial mortar was more than 90% up to a crack width of 400 μm. The limit was three to four times higher than the autogenous healing limit of a mature mortar specimen. Therefore, it can be said that microbial mortars offer self-healing not only for early age cracks but also for the mature cracks.

During the incubation period of 28 days old mortar specimens, available NOx-N concentrations were measured. The consumption of the available NO_3_-N confirmed that the enhanced crack closure performance observed in microbial specimens was due to the microbial activity. Bacterial remains on calcite minerals and mineral formation around the ACDC agglomerates also evidenced the self-healing through MICP.

Crack closure achieved through MICP after 28 days treatment period were comparable with the previously reported microbial self-healing results (Wiktor and Jonkers, 2011; Wang et al., 2014b,c; Silva et al., 2015b). Microbial self-healing studies where ureolytic spores were used as bacterial healing agent reported closure of cracks up to 350–400 μm crack in 28 days (Wang et al., 2014a; Silva et al., 2015b). By incorporating hydrogel protected ureolytic bacterial spores in mortar, more than 80% crack closure up to 485 μm crack width was achieved in 28 days (Wang et al., 2014b). Wiktor and Jonkers (Wiktor and Jonkers, 2011) reported complete crack healing up to 460 μm in 100 days by using expanded clay particles loaded with aerobic-bacterial spores as self-healing additive. The mentioned studies were conducted by using axenic bacterial spores. Incorporation of ACDC appears to give slightly better performance in terms of the healable crack width range and the healing rate. To our knowledge, the only reported trial of non-axenic culture as bacterial agent for microbial self-healing concrete was done with the Cyclic EnRiched Ureolytic Powder (CERUP) (Silva et al., 2015b). Similar to ACDC, CERUP was reported as a self-protected non-axenic culture (Erşan et al., 2015c; Silva et al., 2015b). By incorporating 1% CERUP (CDW/weight cement), the self-healing limit of the mortar specimens could be extended to 450 μm crack width (Silva et al., 2015b). In this study by incorporating 1% ACDC (CDW/weight cement) crack closure up to 500 μm was achieved in the same healing period which is comparable with the reported performance of microbial mortar containing CERUP.

Visual crack closure should always be coupled with other quantification methods to further assess the self-healing performance of mortar specimens (Palin et al., 2015). For example, functionality regain is one of the major interests in self-healing studies (Reinhardt and Jooss, 2003; Van Tittelboom et al., 2011; Li and Herbert, 2012; Wang et al., 2012a,b). So far, strength and water tightness are the common properties being investigated to quantify self-healing efficiency in many studies (Bang et al., 2001; Ramachandran et al., 2001; Van Tittelboom et al., 2011; Li and Herbert, 2012). Considering the initial average crack widths in this study (between 200 and 400 μm), it can be said that significant strength regain is unlikely. The major reason is; the amount of CaCO_3_ produced from the available nutrients was not enough to fill such large cracks and provide strength regain. Since the microbial activity could be followed in this study, one can roughly calculate how much of the crack volume could be completely filled. As results indicated, 14% (102.7 mg) of the NO_3_-N in a mortar specimen (722 mg) became available for microbial consumption and 91% (93.5 mg) of it was reduced by the bacteria at the end of 28 days. In a previous study, the CaCO_3_ precipitation yield of non-axenic cultures were reported as 12.7 g CaCO_3_/g NO_3_-N when Ca(NO_3_)_2_ and NaHCOO were used as nutrients (Erşan et al., 2015a). Based on the reported precipitation yield, the ${\text{NO}}_{3}^{-}$ reduction occurred leads to precipitation of ~1.2 g CaCO_3_. Major form of the CaCO_3_ sealing the cracks was calcite and the density of calcite is 2.7 g/cm^3^. Therefore, the amount of CaCO_3_ produced in this study was enough to fill approximately 440 mm^3^ crack volume. The dimensions of the each specimen were 30 × 30 × 360 mm. The initial average crack width of the 28 days old microbial specimen (containing 0.5% ACDC) was 400 ± 10 μm and it had 4 cracks on the surface. Therefore, one can roughly calculate the total crack volume in the specimen as 1440 ± 36 mm^3^. These values reveal that the microbial produced CaCO_3_ could only fill the 30% of the total crack volume created. Hence for such large crack widths strength regain through MICP is unlikely.

We believe that for concrete durability, protection of the steel reinforcement against corrosion is of significance. In order to protect the steel reinforcement from the aggressive substances, water tightness should be regained to a certain extent following the self-healing of the cracks. Capillary sorption test is one of the ways to quantify the water tightness of the mortar and concrete specimens. In capillary sorption tests conducted, the reference specimen represents the autogenous regain that occurs in regular cracked mortar. The uncracked mortar on the other hand represents the 100% water tightness regain. Therefore, these two values can be considered for evaluation of the water tightness regain through microbial self-healing. Based on the results, microbial self-healing provided 66% (0.5% ACDC) to 74% (1% ACDC) water tightness regain for a tested average crack width of 432 ± 21 μm. Since no capillary sorption experiments were conducted for mature uncracked concrete, water tightness regain could not be calculated for mature specimens. Nevertheless, it is still known that mature microbial specimens absorbed 49–54% less water than the mature control specimens after the healing period. Mentioned major difference between autogenous healing and microbial healing is indicative for a better water tightness regain.

### The healing material and its mechanical properties

In addition to the crack closure ratio, the amount of the healing material and the thickness of the sealing might influence the capillary water absorption. It is reported that autogenous healing mostly occurs at the crack mouth due to the limits in CO_2_ dissolution and related low concentrations inside the crack (Palin et al., 2015). One of the major advantages of microbial healing over autogenous healing is the production of CO_2_ inside the crack. In this process, the activity of the bacteria in the deeper parts of the crack plays a major role. Since O_2_ has dissolution and penetration limits similar to CO_2_, bacterial activity that requires aerobic conditions would be inhibited inside the crack. Contrarily, nitrate (${\text{NO}}_{3}^{-}$) reducing bacteria only rely on presence of ${\text{NO}}_{3}^{-}$ and organic carbon (Erşan et al., 2015a) which could be provided in mortar. Therefore, it was highly possible to observe CaCO_3_ formation inside the crack. Indeed, chemical and physical characterization of the filling material inside the cracks (5 mm < depth < 13 mm) revealed that the control and microbial samples have different compounds. The major compound found inside the crack was CaCO_3_ in microbial samples regardless of their curing age. The found minerals were mostly together with bacterial footprints and bacterial remains which indicated that MICP took place. Visual analysis was further confirmed through EDX and FTIR analysis. Results indicated that inside the cracks of premature control specimens, ettringite and C-S-H were as dominant as CaCO_3_. Contrarily in the powder collected from microbial specimen, forms of CaCO_3_ were the major compounds. In mature control specimens (reference mortar and abiotic control), C-S-H was dominant inside the crack compared to CaCO_3_ (mostly as a result of partial carbonation).

The mechanical properties of the healing materials inside the crack were also tested to elucidate if the microbial induced CaCO_3_ has weaknesses compared to autogenously formed CaCO_3_. It is well known that microbial induced CaCO_3_ has pores/holes due to the growth of the minerals around the bacteria. One of the concerns is a possible decrease in mechanical properties of the microbial calcite due to the porous structure. Weaknesses in mechanical properties may cause detachment or deterioration of the CaCO_3_ minerals which jeopardize the maintenance of functionality regain after self-healing. According to our findings, there is no significant difference between autogenously formed CaCO_3_ and microbial induced CaCO_3_. However, the variation in Martens hardness was higher for microbial induced CaCO_3_ compared to the autogenously formed CaCO_3_. The difference in variation could be attributed to the influence of bacteria related pores. Nevertheless, they did not significantly change the mechanical properties.

To our knowledge, the information about hardness of calcite deposits in cracks is very scarce. Reported hardness values for microbial calcite deposits (between 2.5 and 3 GPa) are comparable with our findings (Xu and Yao, 2014). Regardless of being autogenously formed or microbial induced, the E-modulus values of calcite minerals were between 40 and 50 GPa which were consistent with the reported values for microbial CaCO_3_ deposits (40–50 GPa) (Xu and Yao, 2014).

### The advantages of self-protected non-axenic ACDC culture

The recorded microbial induced self-healing performances by means of a self-protected non-axenic culture appeared to be similar to the previously reported microbial self-healing results where axenic cultures and protective carriers were used. It is reported that the production of the axenic spores becomes expensive for an industrial scale application (Silva et al., 2015a). ACDC is advantageous over reported axenic cultures in many aspects. First of all, the cultivation was done in minimal media which contains concrete admixtures as main nutrients and does not contain trace elements, vitamins, and yeast extract. Therefore, the cost for nutrients could be decreased when compared to the previously reported nutrient solutions for the growth of axenic cultures (Wang et al., 2014c; Silva et al., 2015a). Secondly, the ACDC is a self-protected culture by its layered structure that avoids the need for a protective carrier. Therefore, direct incorporation of the dried ACDC in mortar or concrete is possible. The third advantage is the easy separation of the ACDC from the cultivation media which avoids the centrifugation process. Since ACDC is a type of granular culture with a compact structure, the sludge volume index (SVI) values of the mixture are between 35 and 45 mL/g. Hence, simple settling period of 2–5 min enables to separate ACDC from the liquor. The separated ACDC is ready for drying without further treatment. Eventually, the mentioned advantages play a crucial role to decrease the cost of the healing agent (bacterial agent + nutrients + protective carrier). When the operational expenditure (OPEX) are considered, the production cost of ACDC is about 40 €/kg ACDC most of which is the labor work (See Supplementary Material for calculations). If the reactor and the ACDC quality can be monitored automatically, then the labor work can be decreased by a factor 4 which makes the new OPEX cost range as 17.4 €/kg ACDC (See Supplementary Table 2). When the capital expenditure (CAPEX) of the process is also included in the calculation, total cost of the product becomes 57.4 €/kg ACDC (See Supplementary Material). In this study, 0.5% ACDC in terms of CDW/weight cement (0.71% ACDC/w cement) was found to be enough to achieve a significant microbial induced crack closure in concrete when combined with 3% Ca(NO_3_)_2_ and 2% Ca(HCOO)_2_. Therefore, for an additional cost of ~136 €/m^3^ of concrete, self-healing properties of the concrete can be improved significantly (See Supplementary Table 3). Yet, these are approximate values based on the findings from a 3.2 L scale reactors and tests on lab scale mortar specimens. In order to have a better and brighter picture, further research on optimization and up-scaling of the process is required. The amount of nutrients and the bacterial agent required for a significantly improved self-healing performance should also be optimized to avoid over/under estimations in economic analysis.

### Conclusions

- Combination of ACDC and certain concrete admixtures improved the self-healing capability of the mortar specimens.
- Microbial self-healing by means of the ACDC culture is not only effective for early age cracks but also closes the cracks occurring in mature specimens.
- Incorporation of ACDC (0.5 w/w cement) provided water tightness regain up to 74%.
- Microbial induced CaCO_3_ minerals have similar mechanical properties to the autogenously formed CaCO_3_ minerals when compared through micro-indentation.
- Microbial self-healing with ACDC can be distinguished from the autogenous healing by formation of CaCO_3_ minerals all over the inner crack surface rather than only near the crack mouth.
- Self-protected non-axenic cultures are an economically feasible alternative for development of microbial self-healing concrete.

## Author contributions

YE—The study was held in the context of the author's PhD research on development of microbial self-healing concrete. Experimental design and experimental work, data interpretation, and manuscript preparation was done by this author. EG—Experimental work, data interpretation, and typing of the results was done by this author regarding to the indentation experiments presented for microbial induced CaCO_3_ and autogenously formed CaCO_3_. GL and CL—Significant guidance was provided by these authors during the interpretation of indentation results and revision of the manuscript. Moreover, they have provided the equipment required for the relevant analyses. ND—As the co-supervisor and the corresponding author of the article, she improved the quality of the article and the experimental designs by her expertise on concrete and self-healing phenomena. NB—As the main supervisor of the PhD candidate, he provided significant guidance during the production of the bacterial culture, experimental design, and data interpretation.

### Conflict of interest statement

The authors declare that the research was conducted in the absence of any commercial or financial relationships that could be construed as a potential conflict of interest.
