# Supplementary material for: Self-protected nitrate reducing culture for intrinsic repair of concrete cracks
Source: Front Microbiol. 2015 Nov 4;6:1228. doi: 10.3389/fmicb.2015.01228 (PMC4631954; doi:10.3389/fmicb.2015.01228)
Supplement: Supplementary file 1 [file DataSheet1.DOCX]

Supplementary Material

Self-Protected Nitrate Reducing Culture for Intrinsic Repair of Concrete Cracks

Yusuf Çağatay Erşan^1,2^, Elke Gruyaert^2^, Ghislain Louis^3^, Christine Lors^3^, Nele De Belie^2,^*, Nico Boon^1^

***Correspondence:** Dr. Nele De Belie: nele.debelie@ugent.be

# Supplementary Data

## Calculation of the NO_3_-N leaching percentage from the mortar

The value “14 %” was calculated as follows;

Approximate volume of a batch of a mortar mixture = 1000 cm^3^

Amount of Ca(NO_3_)_2_ in a batch of mortar = 3 % w/w cement = 13.5 g

Amount of NO_3_-N in a batch of mortar = 2.3 g

Dimensions of a specimen 3 cm × 3 cm × 36 cm – reinforcement bar Ø= 6 mm

Approximate volume of one specimen = (3 × 3 × 36) cm^3^ – (Π × 0.3^2^ × 36) cm^3^ = 314 cm^3^

Assumption 1: Homogenous distribution

Amount of NO_3_-N in a specimen = 2.3 g × 314 cm^3^/1000 cm^3^ = 0.722 g NO_3_-N

Percentage passed from mortar to the solution = (0.103 g/0.722 g) × 100 = 14 %

## Calculation of the production cost of ACDC

Calculations in this section are done based on the production of ACDC (kg) in a 1 m^3^ reactor.

### Production yield

ACDC concentration in SBR - 7.8 ± 0.4 g/L TSS and 5.5 ± 0.2 g/L VSS and SVI 45 mL/g

For maintenance of the production VSS ≥ 3 g/L and ACDC harvesting once a week.

Therefore, 2.5 kg ACDC production per week. (~100 L of wet ACDC is collected after the settling period, the cell dry weight (CDW) concentration of wet ACDC is 25 g/L)

### Operational expenditure (OPEX)

#### Nutrient solution

A week of operation requires 28 cycles (4 cycles/day). Volumetric exchange ratio is 50 % (500 L/cycle). Total feed volume required to produce 2.5 kg CDW ACDC is;

0.5 m^3^/cycle × 28 cycles/week × 1 week/2.5 kg CDW ACDC = 5.6 m^3^ nutrient solution/kg ACDC

Assumption 1;

Loading rate of the sequencing batch reactor (SBR) can be decreased by half since there is no need for high loading rate acclimation prior to concrete application. Amount of nutrients required to produce 1 kg of ACDC cost 8.03 € (Table S.1).

#### Pumping and aeration

Influent pump works 4 times/day and 1 h each time. The Grundfos COMFORT (PM**)** pump with 2.5 W energy consumption can serve for such an application. The electricity price for and industrial consumption is 0.1 €/kWh (Silva et al., 2015). Therefore the operation of influent pump costs;

0.0025 kW × 1 hour/cycle × 28 cycles/week × 1 week/2.5 kg ACDC × 0.1 €/kWh = 0.0028 €/kg ACDC

Effluent and ACDC collection can be done by using valves instead of pumps.

Air blower works 3 hours/cycle. Vpuk-TSC 40 air blower can be used to aerate the system. For a capacity of 40 m^3^/h energy consumption is 0.2 kW.

0.2 kW × 3 hour/cycle × 28 cycles/week × 1 week/2.5 kg ACDC × 0.1 €/kWh = 0.67 €/kg ACDC

#### Drying

The water content of the harvested ACDC is 96 %. The water content of the 100 L of wet ACDC (25 g CDW/L) is 96 L. Drying process costs 30 €/m^3^ water evaporated (Silva, 2015). Therefore the drying cost is;

30 €/m^3^ × 0.096 m^3^ water/2.5 kg CDW ACDC = 1.15 €/kg CDW ACDC

#### Labor work

Analyses required to monitor the production process requires 1.5 h/week. A worker earns 50 €/h (Silva, 2015). Therefore, the labor work is;

50 €/h × 1.5 h/week × 1 week/2.5kg CDW ACDC = 30 €/kg CDW ACDC.

If the monitoring is done through automatic measurements, this cost can be decreased by a factor 4. Therefore, it becomes 7.5 €/kg CDW ACDC.

### Capital expenditure (CAPEX)

In order to reach a total cost of a product CAPEX costs should also be considered. One of the typical ways to include CAPEX costs in cost analysis is considering the CAPEX values equal to the OPEX values. Therefore, the total cost of the ACDC becomes 80 €/kg most of which is the labor work. If the reactor and the ACDC quality can be monitored automatically, then the labor work can be decreased by a factor 4 which makes the new OPEX cost range as 17.4 €/kg ACDC but still the CAPEX costs should be kept around 40 €/kg to be able to compensate a cost increase due to the automatization, which makes the total cost as 57.4 €/kg.

## Supplementary Figures

Supplementary Figure 1.: Load cycle for the indentation test

Supplementary Figure 2: Typical load-displacement curve, measured during the indentation test (example for indentation on calcite)


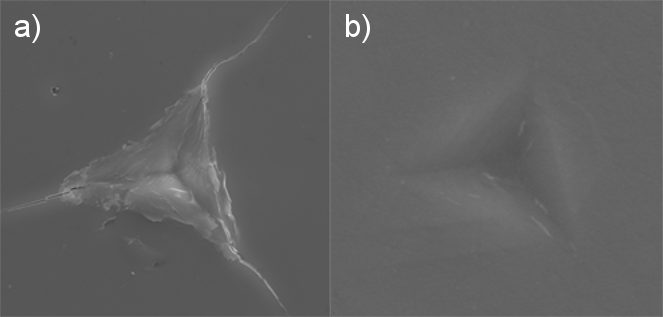


Supplementary Figure 3: Indentation on calcite causing (a) cracks; (b) no cracks

## Supplementary Tables

**Supplementary Table 1. Minimal medium cost calculation for production of ACDC in an SBR**

| **Compounds** | **Concentrations (g/L)** | **Amount required**  **(kg/kg ACDC)** | **Cost^1^**  **(€/ 100 kg)** | **Cost**  **(€/kg ACDC)** |
| --- | --- | --- | --- | --- |
| **NaNO_3_** | 0.85 | 4.76 | 42.5 | 2.02 |
| **NaHCOO** | 2.67 | 14.95 | 38.2 | 5.71 |
| **Ca(HCOO)_2_** | 0.33 | 1.85 | 5.1 | 0.09 |
| **Na_2_HPO_4_.2H_2_O** | 0.03 | 0.17 | 45 | 0.08 |
| **MgSO_4_.7H_2_O** | 0.09 | 0.5 | 26.4 | 0.13 |
| **Total** | - | - | - | 8.03 |
| ^1^Unit prices are based on the quotation (23/07/2015) from BRENNTAG N.V., Belgium | | | | |

**Supplementary Table 2. OPEX costs for production of ACDC for mortar application**

| **Parameter** | **Cost (€/kg ACDC)** | |
| --- | --- | --- |
|  | **Manual monitoring** | **Automated monitoring** |
| Nutrient solution | 8.0 | 8.0 |
| Aeration | 0.7 | 0.7 |
| Pumping | ~0.0 | ~0.0 |
| Drying | 1.2 | 1.2 |
| Labor work | 30 | 7.5 |
| **Sub-total** | **40** | **17.4** |
| CAPEX | 40 | 40 |
| **Total** | **80** | **57.4** |

**Supplementary Table 3. The additives and the additional cost for production of 1 m^3^ microbial self-healing concrete by means of ACDC**

| **Parameter** | **Material cost**  **(€/100 kg)** | **Amount in self-healing**  **concrete^1^ (kg)** | **Cost**  **(€/m^3^ concrete)** |
| --- | --- | --- | --- |
| Ca(HCOO)_2_ | 5.1 | 9 | 0.5 |
| Ca(NO_3_)_2_.4H_2_O | 30.5 | 19.5 | 5.9 |
| ACDC^1^ | ~5740 | 2.25 | 129.2 |
| **Total** |  |  | **135.6** |
| ^1^The amount of ACDC is in terms of cell dry weight content. Addition of 2.25 g bacteria in the form of ACDC requires addition of 3.21 g ACDC since only 70% of the ACDC is bacteria. | | | |

# References

Silva, F. B. (2015). Up-scaling the production of bacteria for self-healing concrete application.

Silva, F., Boon, N., De Belie, N., and Verstraete, W. (2015). Industrial application of biological self-healing concrete: Challenges and economical feasibility. *Commer Biotechnol* 21, 31–38. doi:10.5912/jcb662.
